# Supplementary material for: What do we know about SARS-CoV-2 transmission? A systematic review and meta-analysis of the secondary attack rate and associated risk factors
Source: PLoS One. 2020 Oct 8;15(10):e0240205. doi: 10.1371/journal.pone.0240205 (PMC7544065; doi:10.1371/journal.pone.0240205)
Supplement: S1 Materials — (PDF) [file pone.0240205.s002.pdf]

Supplementary materials – What do we know about SARS-CoV-2 transmission? A systematic review and meta-analysis of the secondary attack rate and associated risk factors

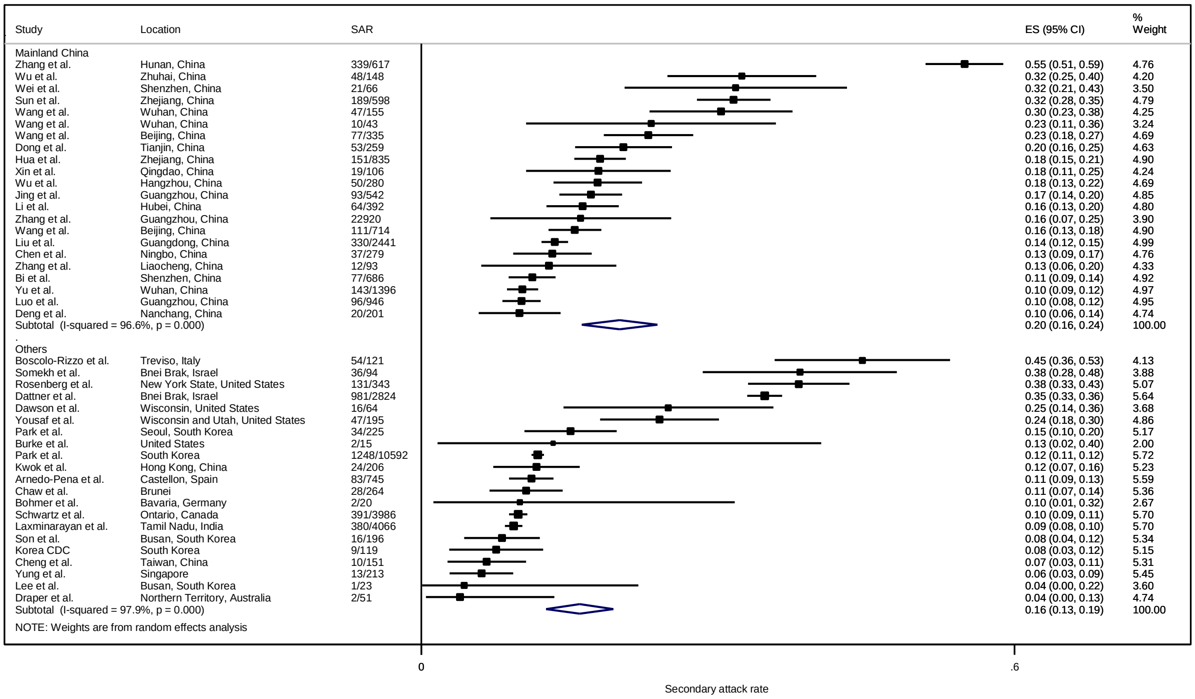

Figure S1. Forest plot of household secondary attack rates (SAR) by location. ES is the estimated SAR, with 95% confidence intervals (CI). I-squared is the percentage of between-study heterogeneity that is attributable to variability in the true effect, rather than sampling variation.

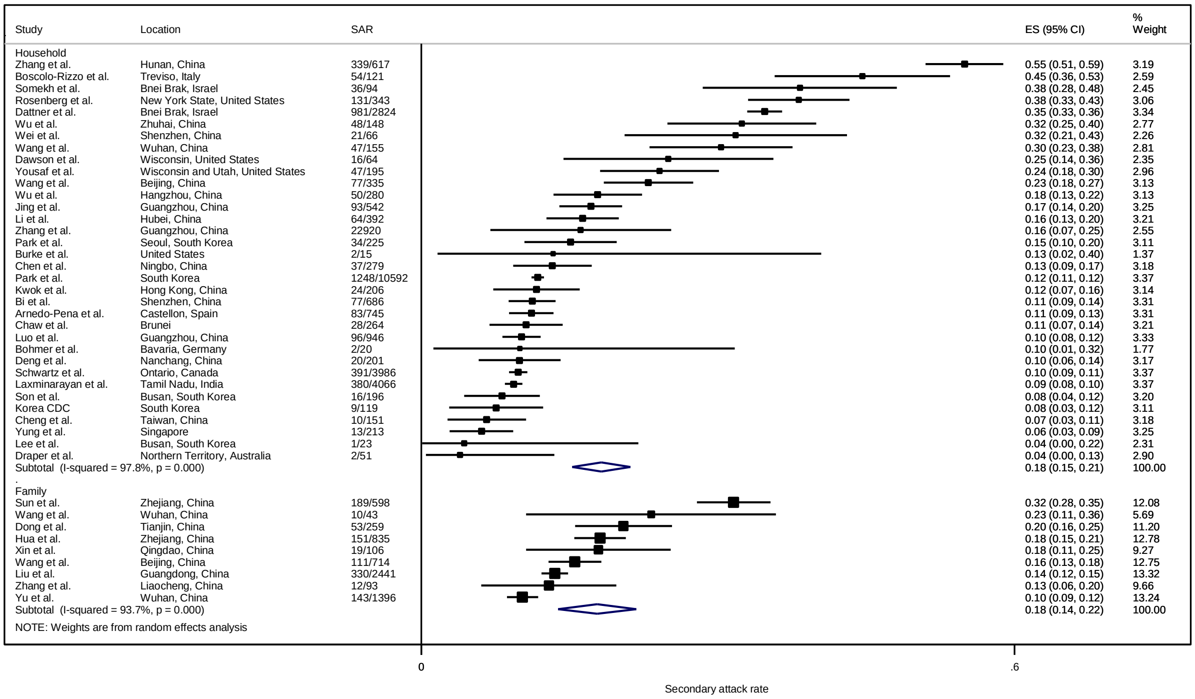

Figure S2. Forest plot of household secondary attack rates (SAR) by definition of close contact. ES is the estimated SAR, with 95% confidence intervals (CI). I-squared is the percentage of between-study heterogeneity that is attributable to variability in the true effect, rather than sampling variation.

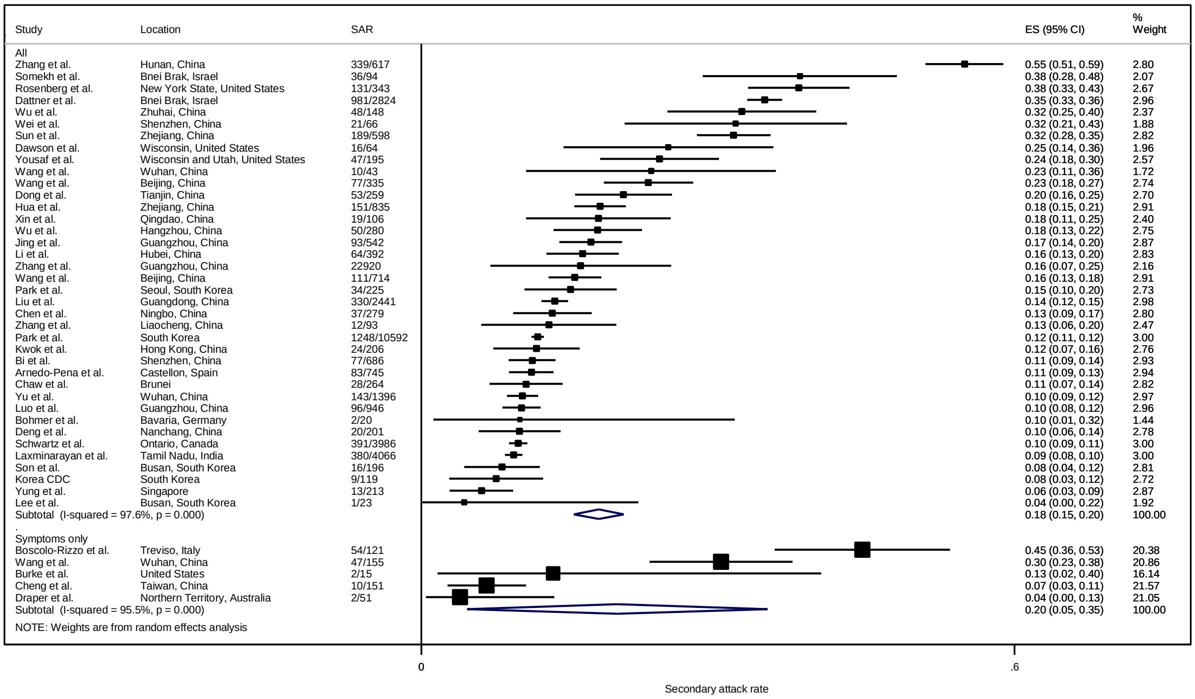

Figure S3. Forest plot of household secondary attack rates (SAR) by testing protocol. ES is the estimated SAR, with 95% confidence intervals (CI). I-squared is the percentage of between-study heterogeneity that is attributable to variability in the true effect, rather than sampling variation.

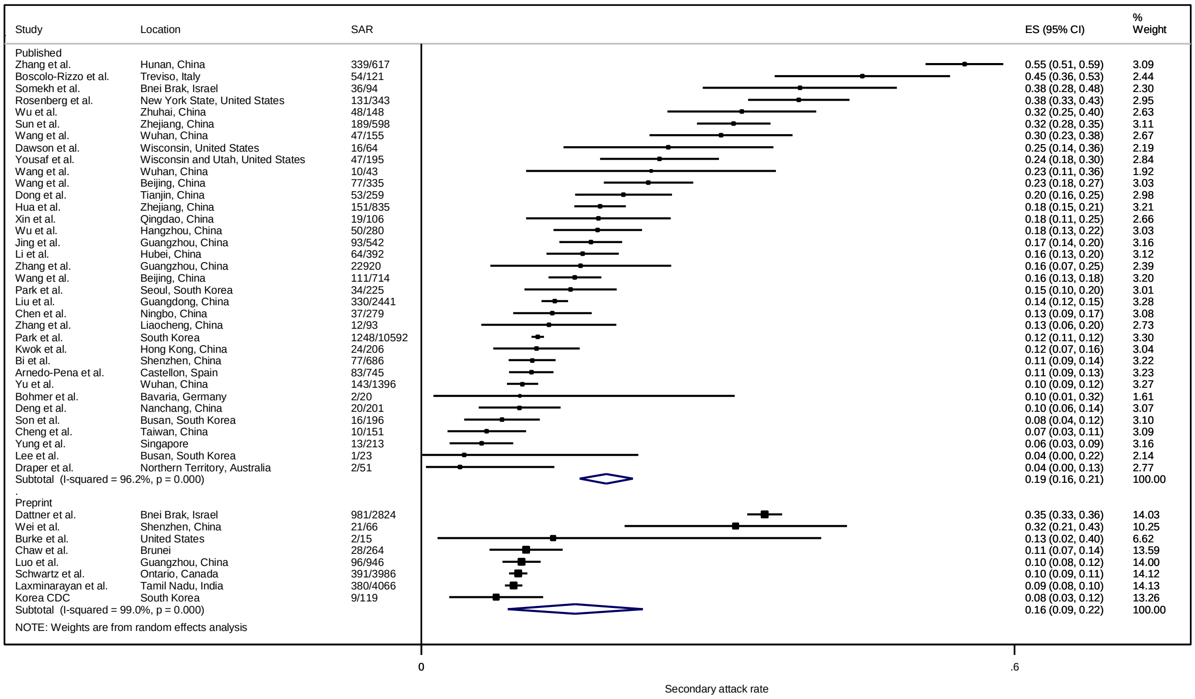

Figure S4. Forest plot of household secondary attack rates (SAR) by publication status. ES is the estimated SAR, with 95% confidence intervals (CI). I-squared is the percentage of between-study heterogeneity that is attributable to variability in the true effect, rather than sampling variation.

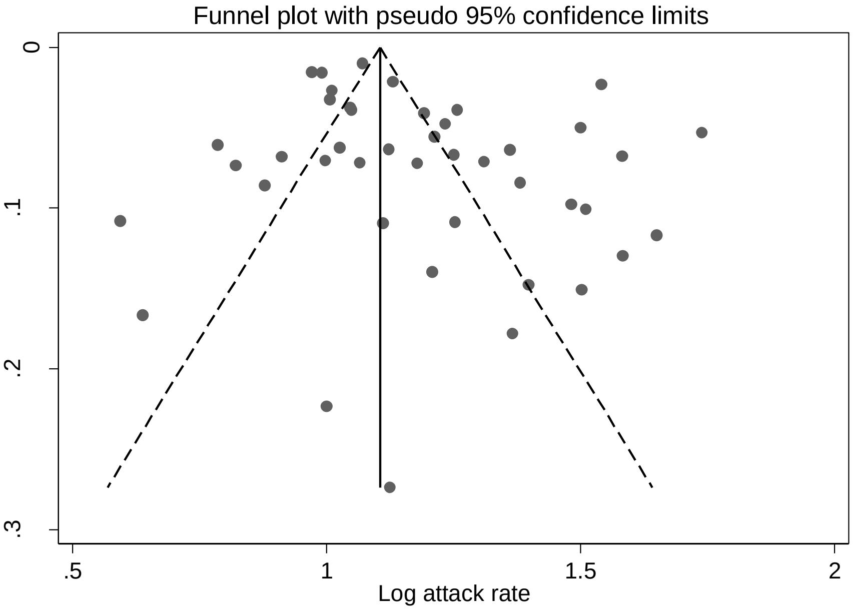

Figure S5. Funnel plot of the 43 studies on household secondary attack rate.

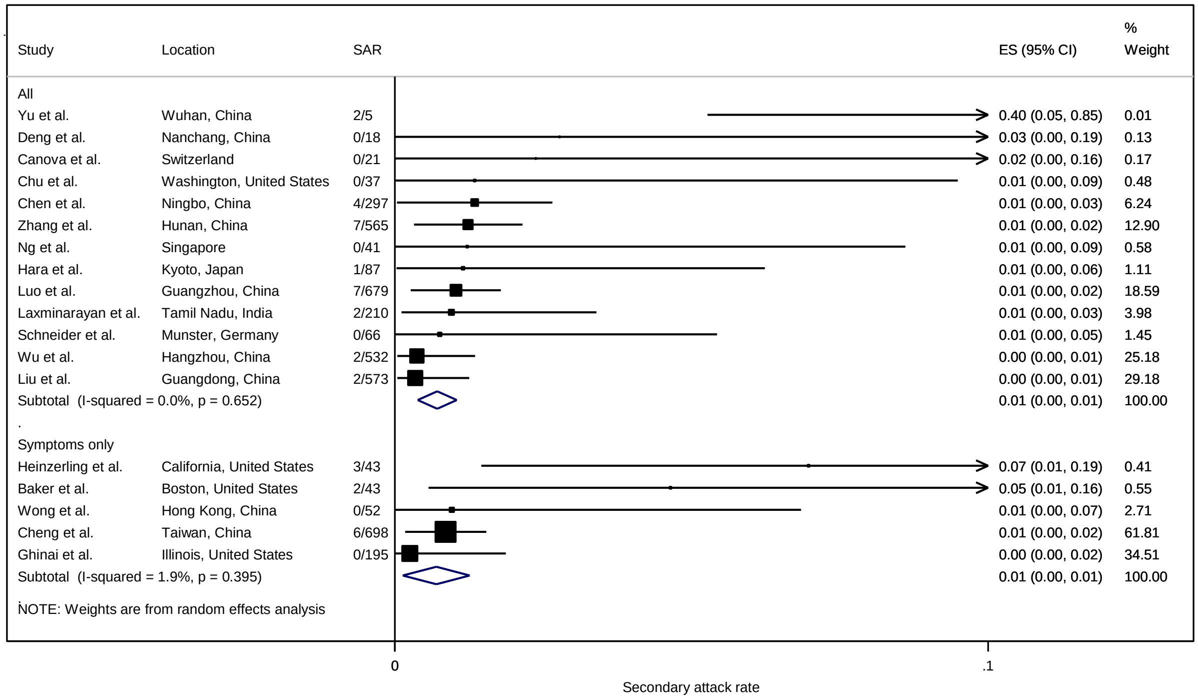

Figure S6. Forest plot of healthcare secondary attack rates (SAR) by testing protocol. ES is the estimated SAR, with 95% confidence intervals (CI). I-squared is the percentage of between-study heterogeneity that is attributable to variability in the true effect, rather than sampling variation.

Parameter	Estimate	SE	t value	p value	95% CI
Slope (coefficient)	1.049	0.039	26.69	0.000	0.970, 1.128
Bias (intercept)	2.086	1.077	1.94	0.060	-0.090, 4.262
Test of H_0: no small-study effects, p value = 0.060					

Table S1. Results from Egger's meta-regression test assessing the presence of publication bias in 43 household secondary attack rate studies

Study	Location	Setting	Attack rate (%)
Feaster and Goh (1)	Pasadena, California	Long-term care facility – residents	408/582 (70.1%)
		Long-term care facility – staff	223/356 (62.6%)
Arons et al. (2)	King County, Washington	Skilled nursing facility	57/89 (64.0%)
Goldberg et al. (3)	United States	Skilled nursing facility	52/97 (53.6%)
Sanchez et al. (4)	Detroit, Michigan	Skilled nursing facility	1207/2773 (43.5%)
Graham et al. (5)	United Kingdom	Nursing home	126/313 (40.3%)
Kimball et al. (6)	King County, Washington	Skilled nursing facility	23/76 (30.3%)
Patel et al. (7)	Illinois	Skilled nursing facility	33/126 (26.2%)
Borras-Bermejo et al. (8)	Barcelona, Spain	Nursing home – residents	768/3214 (23.9%)
		Nursing home – staff	403/2655 (15.2%)
Dora et al. (9)	California	Skilled nursing facility	19/99 (19.2%)
Roxby et al. (10)	Seattle, Washington	Independent and assisted living community	5/142 (3.5%)
Baggett et al. (11)	Boston	Homeless shelter	147/408 (36.0%)
Samuels et al. (12)	Rhode Island	Congregate shelter	35/299 (11.7%)
Ly et al. (13)	France	Homeless shelter	48/683 (7.0%)
Antonio-Villa et al. (14)	Mexico City	Healthcare workers	10925/34263 (31.9%)
Parcell et al. (15)	United Kingdom	Health and social care workers	325/1173 (27.7%)
Lan et al. (16)	Massachusetts	Healthcare workers	83/592 (14.0%)
Lombardi et al. (17)	Lombardy, Italy	Healthcare workers	138/1573 (8.8%)
Barrett et al. (18)	New Jersey	Healthcare workers	40/546 (7.3%)
Jones et al. (19)	United Kingdom	Healthcare workers – symptomatic	38/725 (5.2%)
		Healthcare workers – asymptomatic	52/3644 (1.4%)
Vahidy et al. (20)	Houston, Texas	Healthcare workers – asymptomatic	112/2872 (3.9%)
Luigi et al. (21)	Bari, Italy	Healthcare workers	23/1303 (1.8%)
Ing et al. (22)		Isolated cruise ship	128/217 (59.0%)
Moriarty et al. (23)		Cruise (Diamond Princess)	712/3711 (19.2%)
		Cruise (Grand Princess)	78/469 (16.6%)
James et al. (24)	Arkansas	Church	35/92 (38.0%)
Park et al. (25)	Seoul, South Korea	Call center	94/216 (43.5%)
Lewis et al.(26)	Cabo San Lucas, Mexico	College students in spring break trip	60/183 (32.8%)
Njuguna et al. (27)	Loiusiana	Correctional and detention facility	71/98 (72.4%)
Desmet et al.	Belgium	Daycare centre	0/84 (0%)
Stein-Zamir et al. (28)	Israel	High school – staff	25/151 (16.6%)
		High school – students	153/1161 (13.2%)
Jang et al. (29)	Cheonan, South Korea	Fitness dance class	57/217 (26.3%)
Dyal et al. (30)	United States	Meat processing plant	4913/130578 (3.8%)
Zhang et al. (31)	Shandong, China	Supermarket	11/120 (9.2%)
Yusef et al. (32)	Jordan	Wedding	76/350 (21.7%)

Table S2. Attack rates in selected settings.

References

1. Feaster M, Goh YY. High Proportion of Asymptomatic SARS-CoV-2 Infections in 9 Long-Term Care Facilities, Pasadena, California, USA, April 2020. Emerg Infect Dis. 2020;26(10).
2. Arons MM, Hatfield KM, Reddy SC, Kimball A, James A, Jacobs JR, et al. Presymptomatic SARS-CoV-2 Infections and Transmission in a Skilled Nursing Facility. N Engl J Med. 2020.
3. Goldberg SA, Pu CT, Thompson RW, Mark E, Sequist TD, Grabowski DC. Asymptomatic Spread of COVID-19 in 97 Patients at a Skilled Nursing Facility. J Am Med Dir Assoc. 2020;21(7):980-1.
4. Sanchez GV, Biedron C, Fink LR, Hatfield KM, Polistico JMF, Meyer MP, et al. Initial and Repeated Point Prevalence Surveys to Inform SARS-CoV-2 Infection Prevention in 26 Skilled Nursing Facilities - Detroit, Michigan, March-May 2020. MMWR Morb Mortal Wkly Rep. 2020;69(27):882-6.
5. Graham NSN, Junghans C, Downes R, Sendall C, Lai H, McKirdy A, et al. SARS-CoV-2 infection, clinical features and outcome of COVID-19 in United Kingdom nursing homes. J Infect. 2020.
6. Kimball A, Hatfield KM, Arons M, James A, Taylor J, Spicer K, et al. Asymptomatic and Presymptomatic SARS-CoV-2 Infections in Residents of a Long-Term Care Skilled Nursing Facility - King County, Washington, March 2020. MMWR Morb Mortal Wkly Rep. 2020;69(13):377-81.
7. Patel MC, Chaisson LH, Borgetti S, Burdsall D, Chugh RK, Hoff CR, et al. Asymptomatic SARS-CoV-2 infection and COVID-19 mortality during an outbreak investigation in a skilled nursing facility. Clin Infect Dis. 2020.
8. Borras-Bermejo B, Martínez-Gómez X, San Miguel MG, Esperalba J, Antón A, Martin E, et al. Asymptomatic SARS-CoV-2 Infection in Nursing Homes, Barcelona, Spain, April 2020. Emerg Infect Dis. 2020;26(9).
9. Dora AV, Winnett A, Jatt LP, Davar K, Watanabe M, Sohn L, et al. Universal and Serial Laboratory Testing for SARS-CoV-2 at a Long-Term Care Skilled Nursing Facility for Veterans - Los Angeles, California, 2020. MMWR Morb Mortal Wkly Rep. 2020;69(21):651-5.
10. Roxby AC, Greninger AL, Hatfield KM, Lynch JB, Dellit TH, James A, et al. Detection of SARS-CoV-2 Among Residents and Staff Members of an Independent and Assisted Living Community for Older Adults - Seattle, Washington, 2020. MMWR Morb Mortal Wkly Rep. 2020;69(14):416-8.
11. Baggett TP, Keyes H, Sporn N, Gaeta JM. Prevalence of SARS-CoV-2 Infection in Residents of a Large Homeless Shelter in Boston. JAMA. 2020.
12. Samuels EA, Karb R, Vanjani R, Trimbur MC, Napoli A. Congregate Shelter Characteristics and Prevalence of Asymptomatic SARS-CoV-2. medRxiv. 2020.
13. Ly TDA, Hoang VT, Goumballa N, Louni M, Canard N, Dao ThL, et al. Screening of SARS-CoV-2 among homeless people, asylum seekers and other people living in precarious conditions in Marseille, France, March April 2020. medRxiv. 2020: 2020.05.05.20091934.
14. Antonio-Villa NE, Bello-Chavolla OY, Vargas-Vazquez A, Fermin-Martinez CA, Marquez-Salinas A, Bahena-Lopez JP. Health-care workers with COVID-19 living in Mexico City: clinical characterization and related outcomes. medRxiv. 2020.
15. Parcell B, Brechin K, Allstaff S, Park M, Third W, Bean S, et al. Drive-through testing for SARS-CoV-2 in symptomatic health and social care workers and household members: an observational cohort study in Tayside, Scotland. medRxiv. 2020.
16. Lan FY, Filler R, Mathew S, Buley J, Iliaki E, Bruno-Murtha LA, et al. COVID-19 symptoms predictive of healthcare workers' SARS-CoV-2 PCR results. PLoS One. 2020;15(6):e0235460.
17. Lombardi A, Consonni D, Carugno M, Bozzi G, Mangioni D, Muscatello A, et al. Characteristics of 1573 healthcare workers who underwent nasopharyngeal swab testing for SARS-CoV-2 in Milan, Lombardy, Italy. Clin Microbiol Infect. 2020.

18. Barrett ES, Horton DB, Roy J, Gennaro ML, Brooks A, Tischfield J, et al. Prevalence of SARS-CoV-2 infection in previously undiagnosed health care workers at the onset of the U.S. COVID-19 epidemic. medRxiv. 2020.
19. Jones NK, Rivett L, Sparkes D, Forrest S, Sridhar S, Young J, et al. Effective control of SARS-CoV-2 transmission between healthcare workers during a period of diminished community prevalence of COVID-19. Elife. 2020;9.
20. Vahidy F, Sostman HD, Bernard D, Boom ML, Drews AL, Christensen PA, et al. Prevalence of SARS-CoV-2 infection among asymptomatic healthcare workers in greater Houston: a cross-sectional analysis of surveillance data from a large healthcare system. medRxiv. 2020.
21. Luigi V, Alessandro D, Giovanni M, Luigi M, Antonio C, Marco Q, et al. PREVENTION AND PROTECTION MEASURES OF HEALTHCARE WORKERS EXPOSED IN HEALTH SETTINGS TO SEVERE ACUTE RESPIRATORY INFECTIONS FROM SARS-COV-2 IN A UNIVERSITY HOSPITAL IN BARI, APULIA REGION, SOUTHERN ITALY. J Hosp Infect. 2020.
22. Ing AJ, Cocks C, Green JP. COVID-19: in the footsteps of Ernest Shackleton. Thorax. 2020;75(8):693-4.
23. Moriarty LF, Plucinski MM, Marston BJ, Kurbatova EV, Knust B, Murray EL, et al. Public Health Responses to COVID-19 Outbreaks on Cruise Ships - Worldwide, February-March 2020. MMWR Morb Mortal Wkly Rep. 2020;69(12):347-52.
24. James A, Eagle L, Phillips C, Hedges S, Bodenhamer C, Brown R, et al. High COVID-19 Attack Rate Among Attendees at Events at a Church — Arkansas, March 2020. MMWR Morb Mortal Wkly Rep. 2020.
25. Park SY, Kim YM, Yi S, Lee S, Na BJ, Kim CB, et al. Coronavirus Disease Outbreak in Call Center, South Korea. Emerg Infect Dis. 2020;26(8).
26. Lewis M, Sanchez R, Auerbach S, Nam D, Lanier B, Taylor J. COVID-19 Outbreak Among College Students After a Spring Break Trip to Mexico - Austin, Texas, March 26-April 5, 2020. MMWR Morb Mortal Wkly Rep. 2020;69(26):830-5.
27. Njuguna H, Wallace M, Simonson S, Tobolowsky FA, James AE, Bordelon K, et al. Serial Laboratory Testing for SARS-CoV-2 Infection Among Incarcerated and Detained Persons in a Correctional and Detention Facility - Louisiana, April-May 2020. MMWR Morb Mortal Wkly Rep. 2020;69(26):836-40.
28. Stein-Zamir C, Abramson N, Shoob H, Libal E, Bitan M, Cardash T, et al. A large COVID-19 outbreak in a high school 10 days after schools' reopening, Israel, May 2020. Euro Surveill. 2020;25(29).
29. Jang S, Han SH, Rhee JY. Cluster of Coronavirus Disease Associated with Fitness Dance Classes, South Korea. Emerg Infect Dis. 2020;26(8).
30. Dyal JW, Grant MP, Broadwater K, Bjork A, Waltenburg MA, Gibbins JD, et al. COVID-19 Among Workers in Meat and Poultry Processing Facilities - 19 States, April 2020. MMWR Morb Mortal Wkly Rep. 2020;69(18).
31. Zhang J, Zhuo P, Han D, Wang W, Cui C, Zhou R, et al. Investigation of a cluster epidemic of COVID-19 in a supermarket in Liaocheng, Shandong province. Chinese Journal of Epidemiology. 2020;41.
32. Yusef D, Hayajneh W, Awad S, Momany S, Khassawneh B, Samrah S, et al. Large Outbreak of Coronavirus Disease among Wedding Attendees, Jordan. Emerg Infect Dis. 2020;26(9).
