# Supplementary material for: What do we know about SARS-CoV-2 transmission? A systematic review and meta-analysis of the secondary attack rate and associated risk factors
Source: PLoS One. 2020 Oct 8;15(10):e0240205. doi: 10.1371/journal.pone.0240205 (PMC7544065; doi:10.1371/journal.pone.0240205)
Supplement: Attachment — Submitted filename: SAR_response_to_reviewers.docx [file pone.0240205.s003.docx]

| **REVIEWER 1** |  |
| --- | --- |
| In general, too much information is being forced into one manuscript leading to such high heterogeneity that no real conclusions can be made. I recommend that this be separated into multiple manuscripts in order to find homogeneous populations that can lead to generalizations. For example, it is obvious that SAR would be different in healthcare setting vs. travel vs. people sharing a meal. Lumping these together dilutes the real risk in high-risk settings like sharing a meal. The authors make this same argument in the discussion section, but do not use their recommended nuanced approach in the methods. In this era when COVID guidelines are being written, a “non-household” SAR of 4% may lead to governments stating that it is fine to travel and share meals. The authors need to be very confident in results like this (which they cannot be since there is high heterogeneity) to make a conclusion like that. The conclusions overreach the data and are confusing. We live in a setting where journalists will take conclusions from published abstracts about COVID-19 and use them as headlines in international newspapers. Thus, we need to be extra vigilant that the conclusions match the data. | Thank you for this recommendation.  We agree that there should be separate manuscripts to provide a comprehensive overview with stratified analysis given the heterogeneity observed.  As such, we have focused this first manuscript solely on the secondary attack rate (SAR) to allow for specific groupings of more homogenous populations. We updated the search to obtain more studies and this has allowed us to pool the SAR estimate for we have segregated this into household, healthcare, and other settings.  A second manuscript on the serial interval (SI) and asymptomatic transmission will follow. We aim to submit this second manuscript in the next few weeks. |
| The forest plot in Figure 1 has so much heterogeneity that it is hard to make any conclusions about these studies. a) You should NOT pool “non-household” studies together but rather do subset analyses of more homogenous groups like healthcare settings. b) Since you state that studies used different definitions of household contacts, and your household contact SAR analysis was highly heterogeneous, it would make sense to do a stratified analysis by definition of household contact. c) Also consider stratifying the household SAR analysis into subsets by spouse, child or “other” household members, not as a RR but as a SAR for each group. | We agree with this comment and as such we have amended our meta-analysis on SAR to the following:   - Pooled household SAR and subgroup analyses by stratifying according to location, definition of close contact, testing protocol, and publication status. We also examined whether SAR differed by symptom status of index case, child/adult infection, and relationship of close contacts of index cases. - Pooled SAR in healthcare settings. - 17 studies allowed estimation of SAR in settings or by contact type other than household and healthcare settings. Due to the limited number of studies in each of these settings, unclear or imprecise definitions of close contacts, and the large variation in SAR across the settings, we did not estimate a pooled SAR. Instead, we reported the SAR to highlight potential high-risk settings. |
| Similarly, the forest plot in Figure 3 has so much heterogeneity that it is hard to make any conclusions about these studies. These should also be broken into more homogeneous subsets. | The forest plot in Figure 3 has been removed and the amended analysis which takes into account this comment from Reviewer 1 will be included in the second manuscript on SI and asymptomatic transmission. |
| In every summary (e.g., discussion, abstract) you need to state that the studies were heterogeneous. The included studies are much too different from one another to make any sort of generalizable summary statement. The results section of the abstract should also include the full range of values, not just the 95% confidence intervals, because there is so much heterogeneity. | Thank you for this suggestion.  As there is wide heterogeneity in the household SAR, we have included both the 95% CI and the range in our discussion. |
| This meta-analysis is missing a key component of the PRISMA checklist: Risk of bias in individual studies. Please rate the risk of bias from each study and comment on how the studies with moderate-to-high risk of bias make it difficult for conclusions to be made. There are many risk of bias tools available such as the Institute of Health Economics (IHE) Quality Apprasial of Case Series Studies Checklist Institute of Health Economics, 2014. 2019, at http://www.ihe.ca/research-programs/rmd/cssqac/cssqac-about.) | Thank you for this comment. For the revised manuscript we conducted risk of bias assessment for each individual study identified in the search.  The quality of the studies was independently assessed by three reviewers based on the UK National Institute for Clinical Excellence guidelines (<https://www.nice.org.uk/guidance/cg3/documents/appendix-4-quality-of-case-series-form2>). The evaluation is based on a set of eight criteria. Studies with a score greater than 4 (out of 8) were considered to be of high quality and thus included in the meta analyses. This threshold follows published COVID-19 meta analyses, for example, Zhu et al. (2020) Clinicopathological characteristics of 8697 patients with COVID-19 in China: a meta-analysis. *Fam Med Community Health*. 2020;8(2). |
| Many times, I could not determine whether a subset analysis was done or if the authors just averaged results together without taking into account study weight nor calculating confidence intervals. For example, the asymptomatic proportion of pregnant women needs to be calculated as a meta-analysis with a confidence interval, not just an average of studies. | All the subset analyses were done taking into account study weight with 95% CI.  In the example given on asymptomatic proportion of pregnant women, this has been removed from the revised manuscript and will be included in the second manuscript on SI and asymptomatic infection. |
| The confidence interval calculations for SAR do not make biologic sense. You can’t have a negative SAR or a SAR more than 100%. | We have addressed the negative SAR 95% CI calculations. For data with less than 5 secondary cases, we used a Binomial approximation, which we explicitly mentioned in the Methods section. |
| Potentially missing multiple secondary attack rate studies. | We have updated our literature search to account for all SAR studies up to July 25, 2020. |
| The fitted values in the scatter plots of asymptomatic proportion in the supplement are not very believable since the data points are few and scattered. I would recommend doing a stratified analysis with categorize ages rather than treating them as continuous. | We have removed the scatter plots for asymptomatic proportions in this revised manuscript. This comment from Reviewer 1 will be addressed in the second manuscript on SI and asymptomatic infection. |
| The conclusions overreach the data and are confusing. It doesn’t make sense to say that 10 symptomatic persons living with 100 contacts would result in 15 cases because it would be incredibly rare to have 100 household contacts. Either use a more realistic example or do not use an example like this at all. Similarly, the statement “effective quarantine needs to be implemented within 3 days” does not seem fact based. Where did the number 3 come from? Also, the statements about “targeted physical distancing strategies focusing on high-density enclosed settings” and statements about dormitories and workplaces are not supported by your data because you lump together all non-household interactions. | 10 household contacts for each confirmed case may indeed be rare in Western contexts, however for many countries, large multi-generation households comprising more than 1 nuclear family, are not uncommon ([Data](https://www.un.org/en/development/desa/population/publications/pdf/popfacts/PopFacts_2017-2.pdf) from UN Department of Economic and Social Affairs, Population Division).  In any case, we have removed this illustrative statement. While it is not possible to calculate pooled estimates for all specific setting types identified, due to the limited number of studies, our observations of SAR and AR show a degree of variation with tendency towards higher SAR in institutional and residential settings. |
| I am confused about whether non-English studies were excluded. It is poor practice to exclude studies based on language, especially when an author is from WHO and has access to scientists who speak many languages. | Non-English studies were not excluded, however only the abstract was translated. This point is mentioned in the Methods section, under subheading: Search strategy and inclusion criteria.  In fact, we found several non-English publications through our search and inclusion strategy. For example, for household SAR, there were 5 studies in Chinese language and 2 in Korean language. |
| On page 9, >10 is used when I think you mean <10. | Thank you for spotting this mistake. This refers to the number of studies for SI. Indeed, this is < 10. We will amend accordingly in the second manuscript on SI and asymptomatic infection. |
| Need to say, “publication bias” or “reporting bias” not “small study effect.” | This has been amended. |
| Figure 2 needs to be plotted on a log scale, so the confidence intervals are symmetrical | This refers to the forest plot for SI, which will be included in the second manuscript on SI and asymptomatic infection. |
| I would also recommend performing stratified analysis by publication status. Are the results different among studies that have not yet been peer reviewed compared with peer reviewed studies? | Thank you for this suggestion.  Figure S4 shows a forest plot of household secondary attack rates (SAR) by publication status. There is no significant difference between published and unpublished studies. |
| The discussion section needs to include more limitations. For example, the asymptomatic proportion may be overestimated for the reasons that you state in the results section, but also may be underestimated because people tested may not be representative of the general population | We have amended the limitations section – for limitations on asymptomatic proportion, we will include this recommendation in the second manuscript. |
| Confidence intervals that include 1.00 mean that there is not a statistically significant difference at the p<0.05 level. Please re-word all of your statements about the RR=1.40 comparing adults vs. children since this confidence interval includes 1. | This is well noted. Having updated the search and re-doing the analysis, this is no longer an issue in the new manuscript as the RR for adults vs. children is 1.71 (95% CI: 1.35, 2.17). |
| **REVIEWER 2** |  |
| Overall, the manuscript is clear and well written, and is a systematic look at several important parameters, along with a discussion of their implications. I have a few suggestions to improve the manuscript as it currently stands:  SAR definition: "...dividing the number of exposed close contacts who tested positive (numerator) by the total number of close contacts of the index case (denominator)." Should this not be exposed close contacts for both the numerator and denominator for consistency? Those not at risk (i.e. not exposed) should not be in the denominator. | Thank you for your comments.  For the SAR definition we have altered this to: “SAR……is calculated by dividing the number of exposed close contacts who tested positive (numerator) by the total number of exposed close contacts of the index case (denominator).” |
| Figure S2b: To me, this funnel plot is extremely alarming. At the moment, this is sort of discussed as a side note, but I think it's hard to argue that there's anything other than a pretty systematic bias in terms of the currently published studies on this particular parameter. Given that this is also the parameter that impacts a good deal of the discussion and the implications for policy and interventions, the authors need to spend some more time on this. | This refers to the funnel plot for studies on asymptomatic proportion. This has been removed in the revised manuscript, but we will address this in the second manuscript on SI and asymptomatic infection. |
| Finally, as with all reviews during an emerging epidemic, I suggest a search of the literature between May and now and a re-analysis to ensure that the results are timely as of publication. | Thank you for this suggestion. We have updated the search to include all studies up to July 25, 2020. |
