# Supplementary material for: What do we know about SARS-CoV-2 transmission? A systematic review and meta-analysis of the secondary attack rate and associated risk factors
Source: PLoS One. 2020 Oct 8;15(10):e0240205. doi: 10.1371/journal.pone.0240205 (PMC7544065; doi:10.1371/journal.pone.0240205)
Supplement: Attachment — Submitted filename: 2ndresponse_SAR_Meta_21Sep.docx [file pone.0240205.s004.docx]

| REVIEWER COMMENTS |  |
| --- | --- |
| 1. The meta-analyses shown in Figures 5 and 8 have considerable heterogeneity (the Cochrane Handbook defines considerable heterogeneity as I-squared >75%). When these results are described on pages 9 and 10, sentences need to be added that say “However, there was considerable heterogeneity among the included studies.” | We have added these sentences in our description of the results on p.9 and p.10. |
| 2. The discussion section is overreaching the data. This study did not assess community lockdowns and restricting social movement. Thus, the discussion should not make conclusions about them on page 15 and the final sentence on page 16. It is unknown what the SAR would have been if communities did not restrict social movement when they did. | We have removed the sentences alluding the community lockdowns and movement restrictions on p.15 and p.16. |
| Minor comment page 13: I think there is a typo here and it should say “with a STUDY as well evaluating a religious event” as there were 8 cases at the religious event not one. | Thank you for this comment, we have amended this. |
| Minor comments page 15: 1) I don’t think anxiety is the right word when discussing quarantine fatigue; 2) should be “to better understand their relative susceptibility TO infection” not “of infection.” | Thank you for this comment, we have rephrased this sentence on p.15, and amended (2) |
| ADDITIONAL EDITOR COMMENTS |  |
| 1. Can the authors explain the column header “number of index cases” in more detail in the text, as well add a footnote to the table, particularly as in some cases, the number of index cases is larger than the number of contacts. How was the symptom status of the index case determined when there were multiple index cases? | We have included a description for this on p.6-7 of the manuscript. In general, index cases were confirmed positive cases identified or suspected to have been first exposed to the SARS-CoV-2 virus within the household, generally based on the timing of symptom onset and epidemiological link.  Some of the studies did not specify the number of index cases, which we have marked as “n/a” in Table 1.  As we explained in the “Strengths and limitations” section, the studies are based on contact tracing where the index case determination or the direction of transmission may be uncertain, particularly as a substantial proportion of cases was asymptomatic or mild. |
| 2. Most of the healthcare settings included look at transmission from infected case patients, however a couple of them describe transmission from infected healthcare workers. I would describe those separately, as the latter falls under healthcare workplace “community” transmission, with different mitigation and control measures, whereas the former has implications for risk to healthcare workers during patient care delivery with different risks (e.g., medical procedures) and mitigation via PPE and other protocols. | We take note of this comment and agree that there are likely to be differences in the transmission dynamics from an infected patient vs. a colleague. The former (18 studies) are what are more commonly perceived as true healthcare delivery settings type transmission – with, as noted, important implications for healthcare delivery risks, and the later (2 studies) can also be thought of as a workplace-type setting.  Given this distinction, we have removed the 2 studies that describe transmission from infected healthcare workers from the pooled analysis. |
